# Discordance between trough concentration and tacrolimus exposure on the day of kidney transplantation: a prospective pharmacokinetic study

**DOI:** 10.3389/fphar.2026.1939477

**Published:** 2026-09-14

**Authors:** Hye Eun Kwon, Jin-Myung Kim, Youngmin Ko, Sung Shin, Young Hoon Kim, Joo Hee Jung, Chung Hee Baek, Hyosang Kim, Hyunwook Kwon

**Affiliations:** 1 Division of Kidney and Pancreas Transplantation, Department of Surgery, Asan Medical Center, University of Ulsan College of Medicine, Seoul, Republic of Korea; 2 Division of Nephrology, Department of Internal Medicine, Asan Medical Center, University of Ulsan College of Medicine, Seoul, Republic of Korea

**Keywords:** area under the curve, kidney transplantation, pharmacokinetics, tacrolimus, therapeutic drug monitoring, trough concentration

## Abstract

Tacrolimus trough concentration (C0) serves as a surrogate for drug exposure, but perioperative factors may disrupt this relationship early post-transplantation. We assessed whether the morning trough concentration (C0) on the day of kidney transplantation (POD0) accurately reflects 12-h tacrolimus exposure compared with a relatively stable early reference (POD7). We then derived the POD0 C0 corresponding to the same exposure as the conventional POD7 reference (a C0 of 7–9 ng/mL). We prospectively enrolled 80 *de novo* kidney transplant recipients receiving immediate-release tacrolimus and performed intensive pharmacokinetic sampling on POD0 (C0, C2, C3, C4, C10) and POD7 (C0, C2, C8). Dose-time-anchored AUC0-12 was calculated using the linear trapezoidal rule. The POD0 C0 corresponding to the POD7 reference AUC0-12 was derived by inverting linear, power, and median quantile regression models. Despite only a modest difference in morning C0 values (POD0 median 7.15 vs. POD7 6.40 ng/mL), the POD0 AUC0-12 was substantially higher than that on POD7 (138.9 vs. 105.4 ng·h/mL, p < 0.001); after adjustment for trough concentration, this difference was attenuated but remained significant (approximately +18% at a matched C0). Additionally, the C0–AUC slope on POD0 was approximately 30% lower than on POD7 (12.0 vs. 17.1 per 1 ng/mL). The POD7 reference AUC (125 [IQR 112–144] ng·h/mL at C0 7–9 ng/mL) corresponded on POD0 to a C0 of approximately 6.8 ng/mL (the robust quantile estimate; 5.5–6.8 ng/mL across the three models). Trough C0 levels underestimated tacrolimus exposure on the day of kidney transplantation. On POD0, a trough of approximately 6–8 ng/mL corresponded to an AUC0-12 at or above this relatively stable early reference. These findings are hypothesis-generating and indicate that troughs need not be driven high early to achieve adequate exposure.

## 1 Introduction

Tacrolimus is the cornerstone calcineurin inhibitor in modern kidney transplantation (Staatz and Tett, 2004). Its narrow therapeutic window and significant inter- and intra-patient pharmacokinetic variability necessitate therapeutic drug monitoring (TDM). Contemporary international consensus statements continue to identify the morning whole-blood trough concentration (C0) as the primary monitoring metric in routine clinical practice (Wallemacq et al., 2009; Brunet et al., 2019). Both the second consensus expert panel and current KDIGO guidelines recommend *de novo* target trough ranges, typically 7–9 ng/mL during the first 3 months, calibrated from steady-state pharmacokinetic data (Eckardt et al., 2009; Wallemacq et al., 2009). In practice, physicians use a single morning C0 measurement as a surrogate for the 12-h area under the concentration–time curve (AUC0-12), the exposure metric most directly linked to clinical efficacy and toxicity (Wallemacq et al., 2009; Brunet et al., 2019).

The validity of this surrogate, however, depends on a stable trough–AUC relationship that has been characterized predominantly in steady-state populations (Mardigyan et al., 2005; Wallemacq et al., 2009; Brunet et al., 2019). The first one to three postoperative days are far from steady state. Volatile anesthetics transiently inhibit hepatic CYP3A activity; postoperative ileus and delayed gastric emptying alter absorption; perioperative high-dose methylprednisolone modulates CYP3A and P-glycoprotein activity; and large fluid shifts, coupled with hematocrit changes, alter the whole-blood distribution of tacrolimus (Christians et al., 2006; Naesens et al., 2007; Hesselink et al., 2014; Sikma et al., 2020a; Sikma et al., 2020b). These factors are particularly relevant in deceased-donor kidney transplantation, where the convergence of marginal graft function and excessive calcineurin-inhibitor exposure can prolong delayed graft function and contribute to early tubular injury (Naesens et al., 2007). To our knowledge, however, no prospective study in adult kidney transplant recipients has directly measured tacrolimus AUC on the day of transplantation alongside the same patients' steady-state AUC, so the magnitude of any operative-day mismatch between C0 and AUC has not been quantified.

We therefore performed a prospective, intensive pharmacokinetic cohort study to measure tacrolimus AUC0-12 in the same patients on the day of kidney transplantation (POD0) and 1 week later under stable conditions (POD7). Additionally, we aimed to characterize the relationship between trough concentration (C0) and AUC0-12 at each time point. The objective of this study was to establish the trough concentration target on the day of kidney transplantation that corresponds to equivalent 12-h drug exposure at steady state.

## 2 Materials and methods

### 2.1 Study design and population

This single-center, prospective pharmacokinetic substudy involved *de novo* kidney transplant recipients receiving tacrolimus-based immunosuppression at Asan Medical Center (Seoul, Republic of Korea) between October 2024 and April 2026, conducted as part of the Tacrobell IIT-DOSE study protocol. All recipients received a first, living-donor kidney transplant; there were no deceased-donor recipients and no cases of delayed graft function. Eligible adults were enrolled at the time of transplantation if they were scheduled to receive immediate-release tacrolimus (Tacrobell, Chong Kun Dang Pharmaceutical Corporation, Seoul, Republic of Korea) twice daily and provided written informed consent (Son et al., 2017). The study was approved by the Institutional Review Board of Asan Medical Center (IRB No. 2024-0843) and adhered to the Declarations of Helsinki and Istanbul. Of the 82 enrolled patients, two were excluded from the analytic cohort: one withdrew consent, and one lacked sufficient POD7 sampling, leaving 80 patients for analysis.

### 2.2 Immunosuppression and tacrolimus dosing

All recipients received immediate-release tacrolimus as part of a triple-drug immunosuppressive regimen, which included mycophenolate mofetil or enteric-coated mycophenolate sodium, along with a corticosteroid taper. Induction therapy was administered using basiliximab or anti-thymocyte globulin, following institutional protocols. Tacrolimus was initiated before transplantation per institutional protocol: recipients undergoing desensitization (ABO-incompatible or sensitized recipients) started tacrolimus 5–7 days before transplantation, whereas all other recipients were admitted 2 days before surgery and started tacrolimus with the evening dose (approximately POD-2). Tacrolimus was thereafter administered twice daily at 06:00 and 18:00. Accordingly, the POD0 morning trough represents an early accumulation-phase concentration after several doses, not a first-dose level. Dosages were adjusted by the responsible clinician to maintain a steady-state morning trough concentration (C0) of 7–9 ng/mL, in accordance with current expert recommendations (Eckardt et al., 2009; Wallemacq et al., 2009).

### 2.3 Pharmacokinetic sampling and tacrolimus assay

Intensive pharmacokinetic sampling was conducted on POD0 at five time points: morning C0 immediately before the 06:00 dose, and 2, 3, 4, and 10 h after that dose (designated C2, C3, C4, and C10, respectively). On POD7, sampling occurred at three time points: morning C0, and 2 and 8 h post-dose (C2 and C8). Actual clock times were recorded for each blood draw. Morning C0 samples were also collected daily from POD0 through POD8 to characterize the early-phase exposure trajectory and to anchor the POD1 and POD8 trough levels used for the C12 sensitivity analyses described below. Serum creatinine and the corresponding estimated glomerular filtration rate (eGFR), calculated using the 2021 race-free CKD-EPI creatinine equation (Hwang et al., 2024), were obtained during routine outpatient visits scheduled for POD30, POD60, and POD90; the actual value from the visit day was used regardless of the exact day. All tacrolimus whole-blood concentrations were measured by chemiluminescent microparticle immunoassay (Architect i2000SR, Abbott Laboratories) at the central clinical laboratory of Asan Medical Center.

### 2.4 AUC0-12 calculation and POD0 trough-target derivation

The primary exposure metric was the 12-h area under the tacrolimus whole-blood concentration–time curve (AUC0-12), calculated using the linear trapezoidal rule on a dose-time–anchored time axis, with t = 0 corresponding to the morning dose. For each subject and study day, the AUC over consecutive intervals between sampled time points tᵢ and tᵢ_+1_ was summed as AUC0-12 = Σᵢ ½ × (Cᵢ + Cᵢ_+1_) × (tᵢ_+1_ − tᵢ), where (t_0_, C_0_) equals (0 h, morning trough), and t_last, C_last represents the final intensive sample at 10 h on POD0 or 8 h on POD7. The terminal concentration at 12 h, C (t = 12 h), required to complete the dosing interval, was set equal to the morning C0 of the same period, following the standard steady-state convention used in prior limited sampling studies of tacrolimus (Cantarovich et al., 1998; Op et al., 2007; Benkali et al., 2010; Saint-Marcoux et al., 2010; Niioka et al., 2013; Stifft et al., 2014; Lloberas et al., 2017; Hwang et al., 2024).

Three additional methods for determining the terminal concentration were applied as pre-specified sensitivity analyses and are reported in Supplementary Table S1: linear extrapolation from the last two intensive samples; flat extension (C12 equal to the final observed sample); and a truncated AUC calculated only over the observed sampling window without any extrapolation. Although published Bayesian limited sampling strategies for tacrolimus exist (Op et al., 2007; Mathew et al., 2008; Benkali et al., 2010; Saint-Marcoux et al., 2010; Niioka et al., 2013; Lloberas et al., 2017; Naesens et al., 2024), no published formula matches the specific (C0, C2, C8) sampling structure used at POD7 in our study, and our POD0 protocol uses five rather than three time points. Therefore, direct linear trapezoidal integration on a dose-time anchored axis was used for both days, as it imposes fewer assumptions than a model-based estimator and utilizes every observed concentration.

The C0–AUC0-12 relationship was modeled separately for POD0 and POD7 using three complementary approaches: (i) ordinary least-squares linear regression of the form AUC0-12 = a + b·C0; (ii) a log-log power model (AUC0-12 = a · C0^b) fitted by linear regression on log-transformed data; and (iii) median (50th percentile) quantile regression, which is robust to heteroscedasticity and outliers at the extremes of the C0 distribution.

For each of the three models, the POD0 C0 corresponding to the POD7 reference AUC0-12—defined as the median AUC0-12 of the 26 patients whose POD7 C0 fell within the conventional 7–9 ng/mL range—was obtained by inverting the fitted equation. The three model-specific estimates were reported together, and the convergence (or divergence) of these estimates was used as the primary internal validity check for the derived POD0 target.

### 2.5 Outcomes and statistical analysis

The pre-specified outcomes included tacrolimus AUC0-12 on POD0 and POD7 (primary), the derived POD0 trough target equivalent to the POD7 reference exposure (primary), discharge and 3-month eGFR, and biopsy-proven acute rejection within the first 3 months post-transplant, classified according to the current Banff diagnostic categories (Naesens et al., 2024). Sample size considerations were based on the prospective intensive-sampling protocol of the Tacrobell IIT-DOSE study, which planned 80 paired POD0/POD7 observations to provide ≥80% power to detect a paired AUC0-12 difference of 20 ng·h/mL (approximately a 15% relative change) at α = 0.05, assuming a paired standard deviation of 60 ng·h/mL.

Continuous variables are presented as median [interquartile range (IQR)] and were compared using the Mann–Whitney U test for two groups and the Kruskal–Wallis test for three or more groups. Categorical variables are presented as n (%) and were compared using the chi-square test or, when the expected cell count was less than five, Fisher’s exact test. Within-subject comparisons of paired tacrolimus exposure metrics between POD0 and POD7 were analyzed using the Wilcoxon signed-rank test. The T_max_ distribution was characterized by the mode and the proportion of patients with peak concentration at the canonical 2-h time point.

Spearman’s rank correlation coefficient (ρ) was used to quantify monotonic associations between C0 and AUC0-12. Ordinary least-squares linear regression was employed to estimate slopes, along with 95% confidence intervals and the coefficient of determination (R^2^). The log-log power and median quantile regressions applied during the POD0 target derivation step were fitted in parallel and reported as robustness checks on the linear estimates.

All hypothesis tests were two-sided, with a significance threshold set at 0.05. No correction for multiple comparisons was applied to the pre-specified primary analyses; secondary stratum-by-stratum and sensitivity comparisons are reported as exploratory. All analyses were conducted using Python 3.11.2, with pandas 2.3.3, SciPy 1.17.0, statsmodels 0.14.6, and matplotlib 3.10.8.

## 3 Results

### 3.1 Cohort characteristics

Of the 82 enrolled patients, 80 were included in the analytic cohort (Table 1). The median recipient age was 52 years (IQR, 40–58); 57.5% were male, and 33.8% had diabetes mellitus. The cohort included 26.2% ABO-incompatible transplants, 28.7% desensitization recipients, and induction with basiliximab in 83.8% and anti-thymocyte globulin in 15.0%. When stratified by POD7 C0 (<7, 7–9, >9 ng/mL), no baseline characteristic differed significantly across strata, although there was a borderline trend toward younger age in the 7–9 ng/mL group (median 43.5 vs. 53 years in the <7 group; p = 0.060).

**TABLE 1:** Baseline demographic and clinical characteristics of the 80-patient cohort stratified by POD7 trough C0.

Variable	POD7 C0 <7 (n = 51)	POD7 C0 7–9 (n = 26)	POD7 C0 >9 (n = 3)	p-value
Recipient age, years	53.0 [42.0–60.0]	43.5 [33.5–54.2]	52.0 [41.0–53.0]	0.060
Male sex, n (%)	33 (64.7%)	11 (42.3%)	2 (66.7%)	0.16
Body weight, kg	63.0 [53.5–74.9]	59.9 [52.1–68.4]	67.5 [58.9–70.2]	0.57
Height, m	1.7 [1.6–1.7]	1.6 [1.6–1.7]	1.7 [1.7–1.7]	0.75
Body mass index, kg/m^2^	22.5 [20.8–25.1]	21.9 [19.6–24.0]	21.9 [20.5–23.9]	0.49
Hemodialysis duration, years	5.0 [0.0–14.0]	8.0 [0.8–16.0]	3.0 [1.5–75.0]	0.83
Baseline PRA	4.0 [0.0–24.0]	0.0 [0.0–8.2]	0.0 [0.0–2.0]	0.20
Diabetes mellitus	16 (31.4%)	11 (42.3%)	0 (0.0%)	0.29
Hypertension, n (%)	37 (72.5%)	14 (53.8%)	3 (100.0%)	0.12
ABO-I KT	15 (29.4%)	5 (19.2%)	1 (33.3%)	0.89
Desensitization	17 (33.3%)	5 (19.2%)	1 (33.3%)	0.71
Rituximab (100/200 mg)	11/6 (33.3%)	2/3 (19.2%)	1/0 (33.3%)	0.53
Tacrolimus (CNI)	51 (100%)	26 (100%)	3 (100%)	—
Steroid	50 (98.0%)	26 (100%)	3 (100%)	0.75
Antimetabolite (MMF/Myfortic)	47/4 (100%)	25/1 (100%)	3/0 (100%)	0.71
Induction (Basiliximab/ATG)	44/7 (100%)	21/4 (96.2%)	2/1 (100%)	0.56

Values are median [interquartile range, IQR] for continuous variables and n (%) for categorical variables.

p-values were calculated using the Kruskal–Wallis test (continuous) or chi-square test (categorical).

Abbreviations: POD, post-operative day; C0, trough concentration; PRA, panel reactive antibody; ABO-i KT, ABO, incompatible kidney transplantation; CNI, calcineurin inhibitor; MMF, mycophenolate mofetil; ATG, anti-thymocyte globulin.

### 3.2 Daily exposure pattern (POD0–POD8)

Morning C0 exhibited a characteristic biphasic trajectory over the first eight postoperative days (Figure 1). The median C0 increased from 7.2 ng/mL on POD0 to a peak of 12.7 ng/mL on POD1, declined to a nadir of 5.5 ng/mL on POD4, and stabilized between 6.4 and 6.5 ng/mL on POD7 and POD8. The median total daily tacrolimus dose was 5.5 mg on POD0, reduced to 4.0 mg by POD2 in response to the peak, and then titrated back up to 7.5 mg by POD8. This pattern is consistent with non-steady-state hyperexposure during the first one to three postoperative days, followed by a delayed return toward steady state.

**FIGURE 1:**
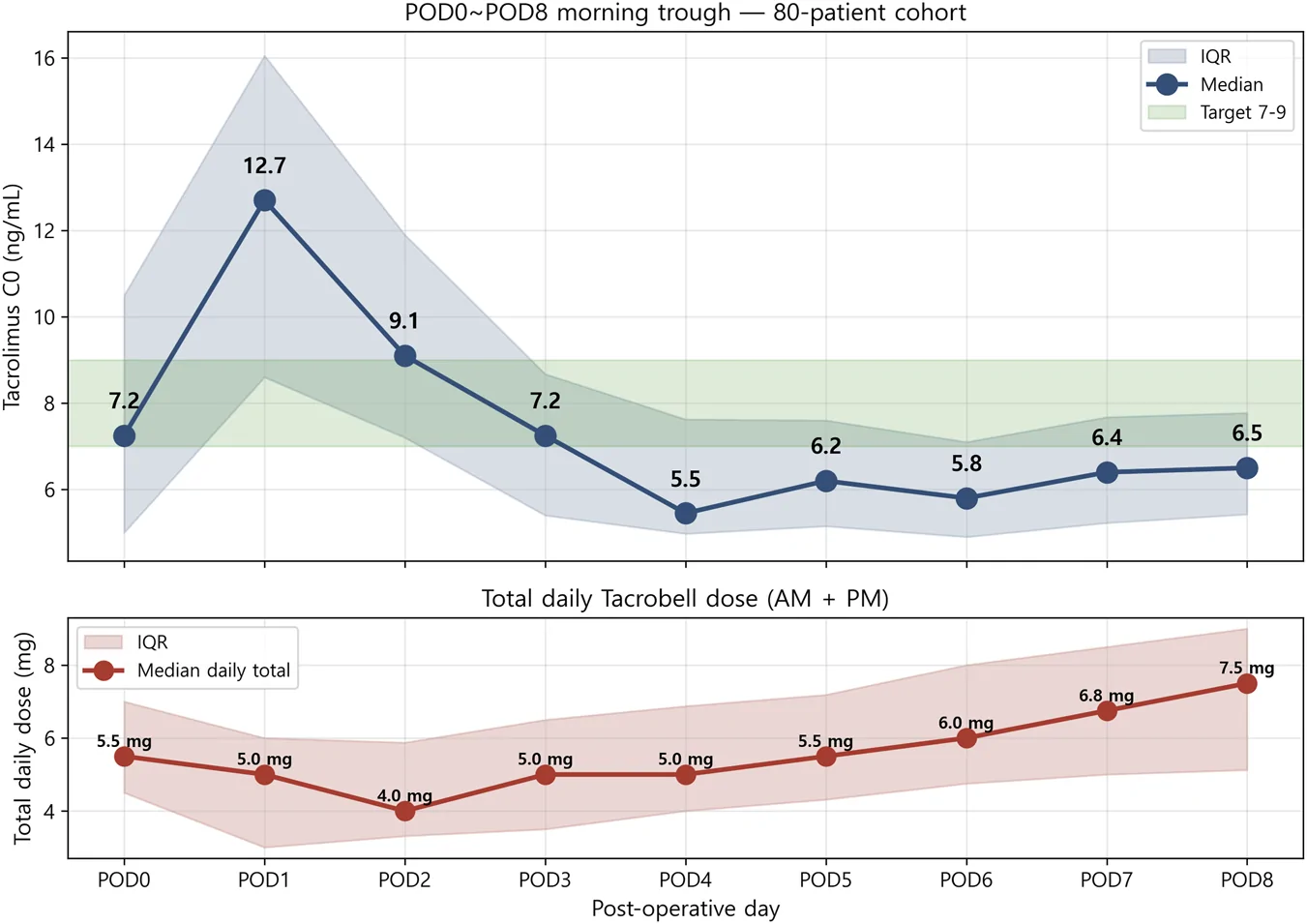
Daily morning trough tacrolimus concentration (C0) and total daily tacrolimus dose from POD0 through POD8 (n = 80). Median C0 (filled circles) and median daily dose (open squares) are shown with IQR. Note the post-operative day 1 peak of approximately 12.7 ng/mL and the subsequent nadir on POD4 before stabilization near the target window. POD, post-operative day; IQR, interquartile range.

### 3.3 Tacrolimus pharmacokinetics on POD0 versus POD7

Despite only a modest, though statistically significant, difference in morning trough concentrations between POD0 and POD7 (median 7.15 vs. 6.40 ng/mL, paired p = 0.013), the full 12-h pharmacokinetic profiles differed substantially between the two time points (Table 2; Figure 2). T_max_ occurred 2 h post-dose in 97.5% of patients on POD0 and 96.2% on POD7. C_max_ was 1.5-fold higher on POD0 than on POD7 (median 20.9 vs. 13.4 ng/mL, p < 0.001), and AUC0-12 was approximately 30% higher on POD0 (median 138.9 [IQR 100.6–205.7] vs. 105.4 [85.2–129.2] ng·h/mL, paired Wilcoxon p < 0.001). Sensitivity analyses for the C12 estimation method produced AUC0-12 values within ±2% of the primary estimate, except for the POD7 linear-extrapolation method (−9.0%) and the observed-window-only method (POD0 -10.9%, POD7 -25.0%) (Supplementary Table S1).

**TABLE 2:** Tacrolimus pharmacokinetic parameters in the 80-patient cohort on POD0 versus POD7.

Parameter	POD0 (acute)	POD7 (stable)	Paired p-value
n	80	80	—
Trough C0, ng/mL	7.15 [5.00–10.50]	6.40 [5.20–7.60]	0.013
C_max_, ng/mL	20.9 [13.9–30.9]	13.4 [10.4–17.2]	<0.001
T_max_ (mode, % of patients)	2 h (97.5%)	2 h (96.2%)	—
AUC0-12, ng·h/mL (primary, SS C12 = C0)	138.9 [100.6–205.7]	105.4 [85.2–129.2]	<0.001
Linear regression: Slope (95% CI)	12.0 (9.4–14.6)	17.1 (12.7–21.5)	—
Coefficient of determination (R^2^)	0.511	0.427	—
Spearman ρ	0.653	0.672	—

Values are median [interquartile range, IQR] unless otherwise indicated. AUC0-12 calculated by linear trapezoidal rule with dose-time anchored t-axis (t = 0 at dose); C12 estimated by steady-state assumption (C12 = C0). Paired p-values from the Wilcoxon signed-rank test.

Abbreviations: POD, post-operative day; C0, trough concentration; C_max_, maximum observed concentration; T_max_, time to maximum concentration; AUC0-12, area under the concentration-time curve over the 0–12 h dosing interval; SS, steady state.

**FIGURE 2:**
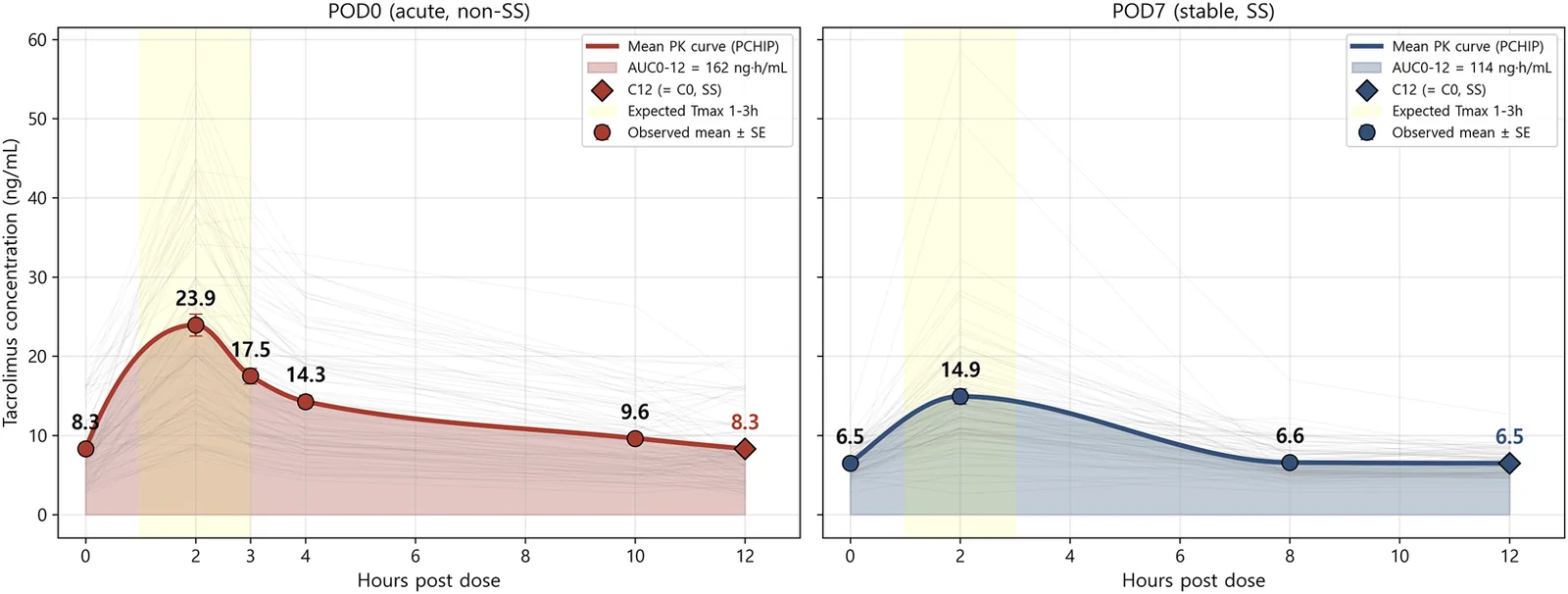
Mean dose-time-anchored 12-h pharmacokinetic profile on POD0 versus POD7 (n = 80). T_max_ was maintained at 2 h post-dose on both days. C_max_ and AUC0-12 were higher on POD0 than on POD7, despite only a modest difference in morning trough concentrations. The shaded area represents AUC0-12, calculated using the linear trapezoidal rule with C12 set equal to the morning C0 based on the steady-state assumption. AUC0-12, 12-h area under the concentration–time curve; T_max_, time to maximum concentration; C_max_, maximum concentration; POD, post-operative day.

### 3.4 Comparison of the trough–AUC relationship between POD0 and POD7

The C0–AUC0-12 relationship was significantly shallower on POD0 than on POD7 (Figure 3; Table 2). Ordinary least-squares linear regression yielded a slope of 12.0 ng·h/mL per 1 ng/mL increase in C0 on POD0 (95% CI, 9.4–14.6; R^2^ = 0.51; Spearman ρ = 0.65; p < 0.001), compared with 17.1 on POD7 (95% CI, 12.7–21.5; R^2^ = 0.43; Spearman ρ = 0.67; p < 0.001). The two 95% confidence intervals were largely non-overlapping, with the upper bound of the POD0 slope below the POD7 point estimate, indicating that the approximately 30% reduction in slope on the day of surgery is robust to sampling variability. The log-log power model on POD0 (AUC0-12 = 40.6 × C0^0.63^) was sublinear, indicating saturation at the higher end of the observed C0 range, whereas the POD7 model was closer to proportional.

**FIGURE 3:**
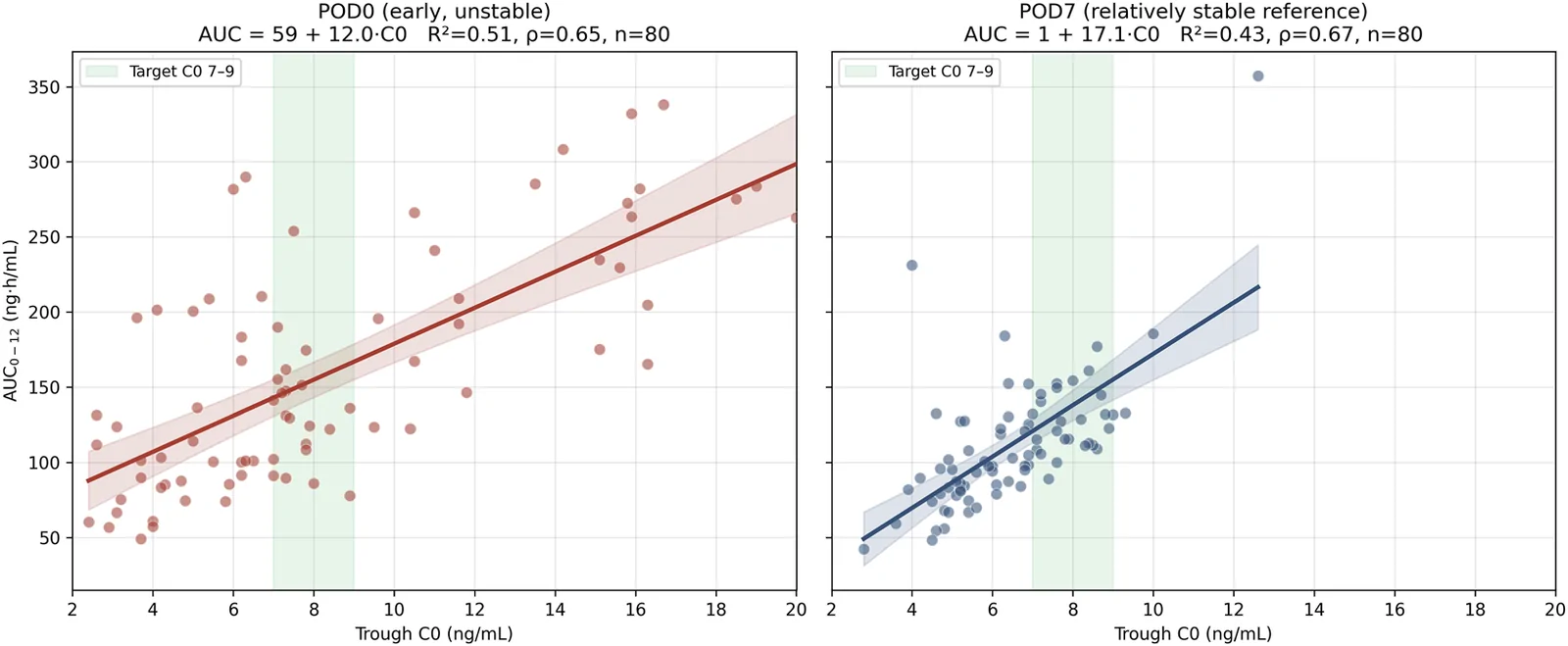
Scatter plot and linear regression of trough concentration versus AUC0-12 on POD0 (red) and POD7 (blue) (n = 80). The slope on POD0 was approximately 30% lower than that on POD7 (12.0 vs. 17.1 ng·h/mL per 1 ng/mL increase in C0). Solid lines represent ordinary least-squares linear regression; shaded bands indicate 95% confidence intervals.

### 3.5 AUC0-12 by C0 stratum

Stratification by POD7 C0 revealed a clear dose-response relationship in AUC0-12: 93.4 [79.2–106.5] ng·h/mL in the <7 ng/mL group, 125.0 [111.8–143.8] ng·h/mL in the conventional 7–9 ng/mL group, and 185.7 [159.3–271.4] ng·h/mL in the >9 ng/mL group (Kruskal–Wallis p < 0.001). Stratification by POD0 C0 showed a similar pattern but with substantially higher absolute AUC values (237.9 [188.0–277.0] ng·h/mL in the >9 ng/mL POD0 group), confirming that the same C0 corresponds to greater drug exposure on POD0 (Supplementary Table S3). The conventional POD7 7–9 ng/mL reference AUC of 125 ng·h/mL falls within Stifft’s published maintenance AUC range of 100–150 ng·h/mL and at the lower margin of the European consensus *de novo* target range (130–200 ng·h/mL) (Figure 4) (Mardigyan et al., 2005; Wallemacq et al., 2009; Stifft et al., 2014).

**FIGURE 4:**
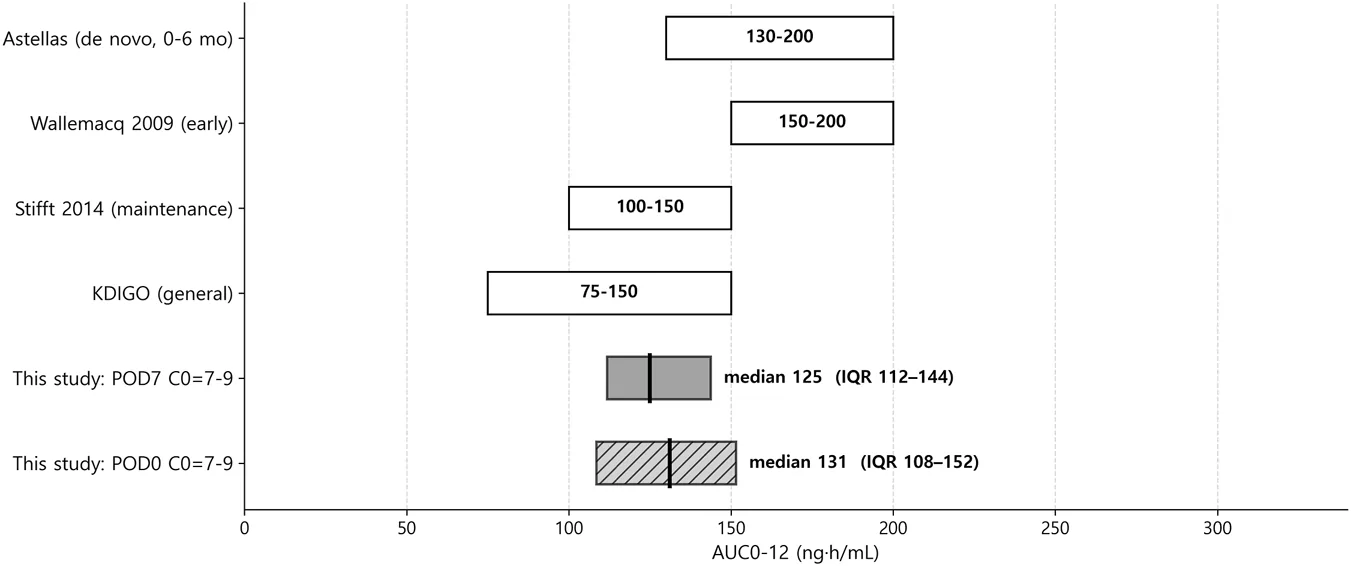
Published target AUC0-12 ranges compared with the present cohort’s POD7 reference exposure. The POD7 AUC0-12 corresponding to a trough level of 7–9 ng/mL was 125 [IQR 112–144] ng·h/mL, which falls at the lower margin of the *de novo* European consensus target range (130–200) and within the Stifft maintenance band (100–150). POD, post-operative day.

### 3.6 Operative-day C0 target equivalent to the steady-state reference

When inverted at the POD7 reference AUC of 125 ng·h/mL, the three independent regression models converged on a POD0 trough target of approximately 5.5–6.8 ng/mL (linear: 5.5 ng/mL; power: 5.9 ng/mL; median quantile regression: 6.8 ng/mL) (Figure 5; Supplementary Table S2). The corresponding range for the POD7 reference IQR of 112–144 ng·h/mL was 4.4–8.3 ng/mL. These model-based estimates should be interpreted with caution: the linear value (5.5 ng/mL) is strongly influenced by a high-C0 tail, whereas the robust quantile estimate (∼6.8 ng/mL) lies at the lower edge of the conventional range. We therefore regard these figures as hypothesis-generating rather than as a revised target.

**FIGURE 5:**
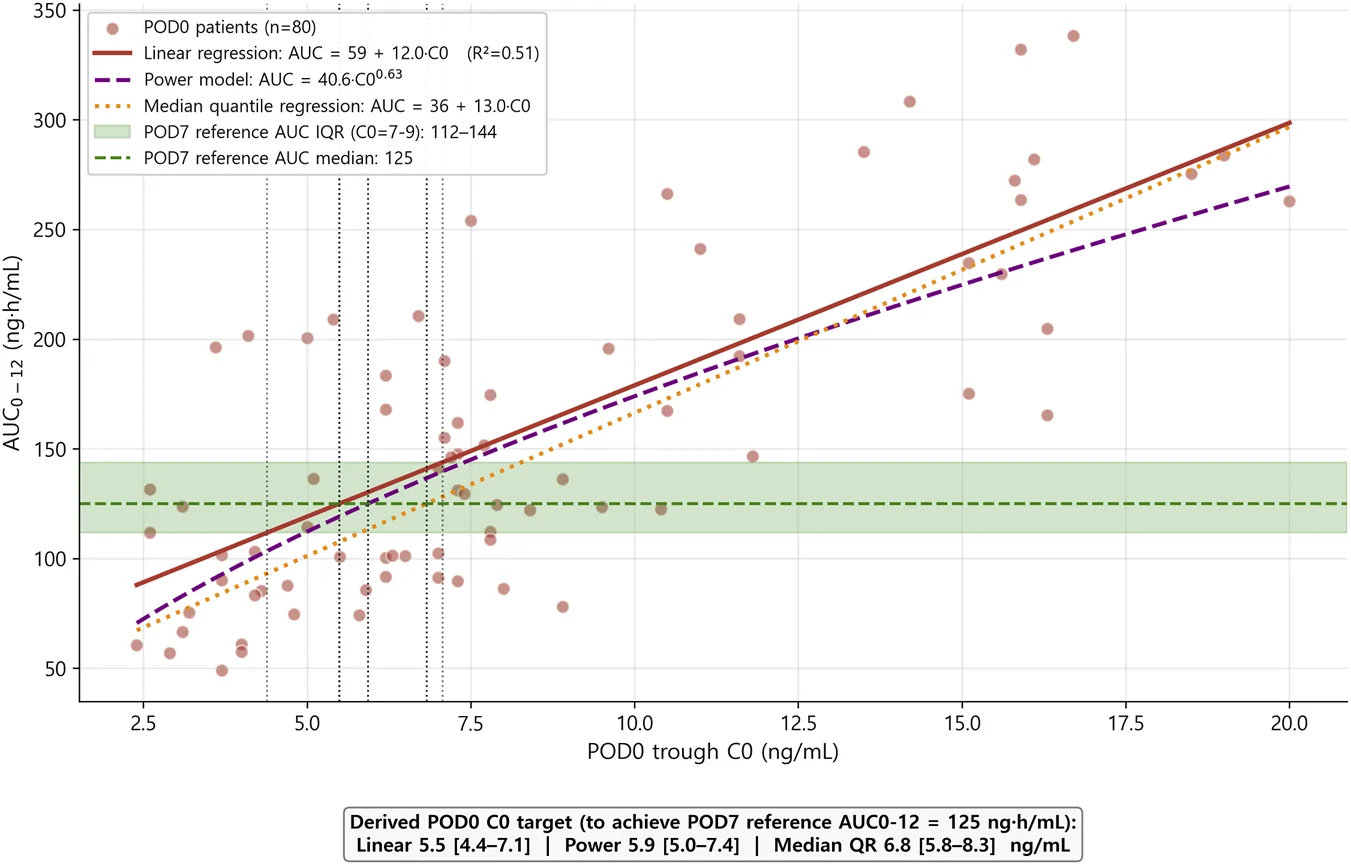
Derivation of the POD0 trough concentration target equivalent to the POD7 reference AUC0-12 using three regression models. Each fitted curve (linear, power, and median quantile regression) is inverted at the POD7 reference AUC0-12 value of 125 ng·h/mL (IQR 112–144). The three independent estimates converge on a POD0 trough target range of 5.5–6.8 ng/mL.

### 3.7 Early renal function and rejection

Discharge eGFR (median 75.0 [63.7–95.7] mL/min/1.73 m^2^) and 3-month eGFR (median 63.0 [53.5–78.6] mL/min/1.73 m^2^) did not differ significantly across POD7 C0 strata (p = 0.614 and 0.625, respectively; Table 3). Two of 80 patients (2.5%) underwent a rejection-suspected biopsy through POD120 (one in the <7 group, one in the 7–9 group; no events in the >9 group; p = 0.853). Pathology adjudication was not yet finalized at the analysis cut-off.

**TABLE 3:** Three-month post-transplant renal function and rejection events stratified by POD7 trough C0.

Variable	POD7 C0 <7 (n = 51)	POD7 C0 7–9 (n = 26)	POD7 C0 >9 (n = 3)	p-value
Discharge eGFR (mL/min/1.73m^2^)	74.4 [63.1–92.6]	80.3 [64.0–99.0]	64.6 [64.4–69.8]	0.61
Discharge serum Cr (mg/dL)	1.02 [0.83–1.29]	0.92 [0.82–1.31]	1.17 [1.10–1.33]	0.55
3-month eGFR (mL/min/1.73m^2^)	64.3 [50.5–75.2]	67.0 [56.0–85.0]	61.5 [60.8–62.2]	0.63
3-month serum Cr (mg/dL)	1.21 [0.99–1.54]	1.06 [0.89–1.40]	1.38 [1.22–1.43]	0.46
Rejection-suspected event^†^, n (%)	1/51 (2.0%)	1/26 (3.8%)	0/3 (0.0%)	0.85

^†^Rejection-suspected events indicate patients who underwent allograft biopsy for suspected rejection through POD120.

Abbreviations: POD, post-operative day; eGFR, estimated glomerular filtration rate; CKD-EPI, chronic kidney disease epidemiology collaboration; BPAR, biopsy-proven acute rejection.

## 4 Discussion

In this prospective intensive pharmacokinetic study involving 80 *de novo* kidney transplant recipients, we found that the morning trough C0 on the day of transplantation significantly underestimated the 12-h tacrolimus exposure compared to the steady-state day. The slope of the C0–AUC0-12 relationship was approximately 30% lower on POD0 than on POD7, and three independent regression methods consistently indicated an equivalent-exposure POD0 trough of approximately 6–7 ng/mL (robust estimate ∼6.8 ng/mL, at the lower edge of the conventional 7–9 ng/mL range)—to achieve an AUC0-12 equivalent to the steady-state reference. These results provide quantitative evidence that conventional trough-based dose titration may underestimate exposure on the day of surgery; after adjustment for trough concentration, however, this difference was modest (approximately +18% at a matched C0; Supplementary Table S4), and we therefore interpret the equivalent-exposure estimates cautiously.

Several studies have characterized the C0–AUC relationship and developed limited sampling strategies (LSSs) for tacrolimus, almost all in steady-state populations (Cantarovich et al., 1998; Mardigyan et al., 2005; Op et al., 2007; Mathew et al., 2008; Benkali et al., 2010; Saint-Marcoux et al., 2010; Alloway et al., 2012; Stifft et al., 2014). The Wallemacq consensus proposed a *de novo* AUC0-12 target of 130–200 ng·h/mL (Wallemacq et al., 2009), while Stifft and colleagues reported a maintenance target of 100–150 ng·h/mL with reduced variability under once-daily formulations (Stifft et al., 2014). Population pharmacokinetic analyses using Bayesian estimators have refined individualized dose prediction but have similarly used post-discharge sampling (Op et al., 2007; Benkali et al., 2010; Saint-Marcoux et al., 2010). Our POD7 reference AUC of 125 ng·h/mL at C0 7–9 ng/mL falls within the Stifft maintenance range and at the lower end of the *de novo* target, supporting the external validity of our early reference measurement. On POD0, the same patients exhibited a markedly higher C_max_ and approximately 30% higher AUC0-12 at virtually the same morning trough, with a sublinear C0–AUC relationship (power exponent 0.63) indicating exposure saturation at higher trough levels. This operative-day deviation from the relatively stable early reference has not previously been quantified in adult kidney transplant recipients.

The derivation of the operative-day trough target involved four steps rather than a single calculation. First, the steady-state reference AUC0-12 was anchored to the 26 patients whose POD7 C0 fell within the 7–9 ng/mL range. Second, separate POD0 and POD7 C0–AUC0-12 models were fitted using three statistical frameworks. Third, each model was inverted at the reference AUC to yield a model-specific POD0 C0. Fourth, the three estimates were compared for convergence, resulting in a robust range of 5.5–6.8 ng/mL. The convergence of linear, power-law, and median quantile regression estimates—each based on different assumptions about variance structure and linearity—provides the strongest internal support for the proposed target. The clinical relevance of avoiding operative-day overexposure is supported by prior studies linking early high tacrolimus exposure to subclinical tubular injury and subsequent interstitial fibrosis and tubular atrophy (Naesens et al., 2007), to early neurotoxicity and nephrotoxicity in thoracic transplant recipients (Sikma et al., 2020a; Sikma et al., 2020b), and the early phase characterized by pronounced intrapatient exposure variability (Ekberg et al., 2011; Shuker et al., 2016; Süsal and Döhler, 2019). Therefore, our findings offer a quantitative basis for operative-day dose reduction beyond the qualitative recognition that the first postoperative days are unstable.

The clinical importance of avoiding early tacrolimus overexposure is particularly pronounced in deceased-donor kidney transplantation, where delayed graft function (DGF) is common and serves as an independent risk factor for acute rejection and reduced long-term graft survival (Lim et al., 2019). In this context, calcineurin inhibitor (CNI) nephrotoxicity, superimposed on ischemia–reperfusion injury, can prolong DGF and exacerbate early tubular damage. This provides the rationale for several clinical strategies aimed at minimizing or delaying CNI exposure during the initial postoperative days. Nair and colleagues demonstrated that an induction protocol combining antithymocyte globulin (ATG) with delayed CNI initiation can preserve early renal function in recipients at high risk for DGF, effectively withholding tacrolimus during the operative window when overexposure would be most harmful (Nair et al., 2021). Beyond outright CNI delay, early postoperative conversion to belatacept has improved renal function recovery among recipients with prolonged DGF by eliminating calcineurin inhibitor exposure during this vulnerable phase (Wojciechowski et al., 2017). The willingness of contemporary protocols to withhold or substitute tacrolimus altogether to avoid acute overexposure underscores the clinical significance of operative-day exposure overshoot. Because our cohort comprised exclusively living-donor recipients without DGF, these strategies lie outside our clinical setting and are cited only as context. Our data contribute the previously missing early-perioperative exposure information relevant to such settings, showing that on the operative day an adequate AUC0-12 is achieved at moderate troughs. The accumulated evidence linking early high tacrolimus exposure to histologic and functional decline (Naesens et al., 2007; Niioka et al., 2013; Sikma et al., 2020a; Sikma et al., 2020b) suggests that avoiding unnecessary early over-exposure warrants further prospective study.

Several biological mechanisms plausibly underlie the day-of-surgery departure from steady-state pharmacokinetics. Volatile anesthetics transiently inhibit hepatic CYP3A activity, and perioperative high-dose corticosteroids can modulate both CYP3A and P-glycoprotein–mediated efflux (Christians et al., 2006; Hesselink et al., 2014; Birdwell et al., 2015). Acute fluid shifts and rapid hematocrit changes alter whole-blood tacrolimus distribution (Sikma et al., 2020b). Importantly, in our cohort, T_max_ was preserved at 2 h post-dose in over 96% of patients on both POD0 and POD7, indicating that the timing of absorption was not appreciably delayed; rather, the extent of absorption—reflected in the higher C_max_ and AUC0-12—was increased on POD0. Prior strategies for early-phase dose individualization have focused on pre-transplant CYP3A5 genotyping (Hesselink et al., 2010; Hesselink et al., 2014; Birdwell et al., 2015; Kim et al., 2021), with the Clinical Pharmacogenetics Implementation Consortium publishing guidelines for CYP3A5 genotype-guided tacrolimus dosing (Kim et al., 2021). To our knowledge, however, no prior strategy has proposed a quantitative operative-day trough target informed by direct AUC measurement on the same patients’ steady-state day. Our findings provide an evidence base for such an approach that is complementary to, rather than dependent on, pharmacogenetic information.

Two practical aspects of the sampling design warrant discussion. First, the POD0 intensive sampling protocol included five time points, whereas POD7 included three. This asymmetry reflected clinical reality: on POD0, the patient was already undergoing intra-operative arterial blood gas analysis and recovery-room laboratory draws, so additional timed samples could be obtained without significant additional burden; on POD7, the patient was on the general ward, making multiple additional venipunctures impractical. To compensate for the sparser POD7 design, the AUC0-12 was calculated using the linear trapezoidal rule on a dose-time-anchored time axis, with the terminal C12 set equal to the morning trough under the standard steady-state assumption (Cantarovich et al., 1998; Stifft et al., 2014). Four sensitivity analyses for the C12 estimation method—primary steady-state, linear extension, flat extension, and observed-window-only—produced AUC0-12 values within ±2% of the primary estimate (except the POD7 linear-extrapolation method, −9.0%), and Tmax timing (96%–97% at 2 h) was consistent with the published immediate-release tacrolimus profile (Staatz and Tett, 2004; Stifft et al., 2014). The convergence of three regression models on a 5.5–6.8 ng/mL POD0 target, the consistency of the POD7 reference AUC with prior maintenance ranges (Stifft et al., 2014), and the robustness of the AUC0-12 estimates across alternative C12 assumptions collectively support the accuracy of our exposure calculations. Compared with prior LSS studies, our approach shares the linear trapezoidal AUC framework but introduces dose-time anchoring on POD0 with intensive five-point sampling, reducing reliance on steady-state assumptions that have limited the extension of LSS to the operative day (Op et al., 2007; Mathew et al., 2008; Benkali et al., 2010; Saint-Marcoux et al., 2010; Niioka et al., 2013; Lloberas et al., 2017).

This study has several limitations. First, it was conducted at a single academic center using only one immediate-release tacrolimus formulation; therefore, extending the findings to once-daily extended-release formulations and other centers will require external validation. Second, the POD7 C0 > 9 ng/mL stratum included only three patients, resulting in considerable uncertainty around the upper end of the C0–AUC curve. Third, the mechanistic origins of the POD0–POD7 pharmacokinetic changes—including the relative contributions of CYP3A inhibition, P-glycoprotein modulation, postoperative ileus, and hematocrit changes—were not directly investigated in this study and warrant dedicated mechanistic research. Fourth, POD7 does not represent true steady state; the concentration/dose ratio continued to decline through POD8 (Supplementary Figure S1), so POD7 is best regarded as a relatively stable early reference, and true steady-state exposure (at approximately 1–3 months) was not sampled. Fifth, tacrolimus was initiated before transplantation, with heterogeneous pre-transplant exposure (5–7 days in desensitized/ABO-incompatible recipients versus approximately 2 days in others); although the exposure discordance persisted in both subgroups (Supplementary Table S6), this heterogeneity should be considered. Sixth, POD0 and POD7 used asymmetric sampling (five versus three timepoints); a matched three-point analysis preserved the difference (Supplementary Table S5). Seventh, CYP3A5 genotype/activity—a major determinant of tacrolimus pharmacokinetics—was not measured, and the cohort was an exclusively living-donor, Asian population; geographic, racial, and pharmacogenetic differences limit generalizability.

## 5 Conclusion

Morning trough tacrolimus concentrations underestimated 12-h drug exposure on the day of kidney transplantation, with a trough–AUC slope approximately 30% lower than at the relatively stable early reference. On POD0, a moderate trough (approximately 6–8 ng/mL) already corresponded to exposure at or above this relatively stable early reference. We present the equivalent-exposure estimates as hypothesis-generating rather than as a revised dosing target. Future work should include prospective multicenter validation, particularly in deceased-donor recipients at high risk of delayed graft function; a randomized comparison of the conventional 7–9 ng/mL target versus the proposed 5.5–6.8 ng/mL POD0 target, using delayed graft function recovery and biopsy-proven rejection as endpoints; and integration of the operative-day target with CYP3A5 genotype-guided dosing and Bayesian population pharmacokinetic estimators to enable individualized POD0 dose tailoring.

## Data Availability

The raw data supporting the conclusions of this article will be made available by the authors, without undue reservation.
