# Supplementary material for: Discordance between trough concentration and tacrolimus exposure on the day of kidney transplantation: a prospective pharmacokinetic study
Source: Front Pharmacol. 2026 Sep 14;17:1939477. doi: 10.3389/fphar.2026.1939477 (PMC13616799; doi:10.3389/fphar.2026.1939477)
Supplement: Supplementary file 1 [file Supplementaryfile1.docx]

**Supplementary Tables**

**Supplementary Table S1. Sensitivity analysis of AUC0–12 by C12 estimation method.**

| **Period** | **C12 estimation method** | **n** | **Median [IQR]** | **Mean ± SD** | **% diff from primary** |
| --- | --- | --- | --- | --- | --- |
| POD0 (acute, non-SS) | SS (C12 = C0, primary) | 80 | 138.9 [100.6–205.7] | 158.5 ± 75.1 | +0.0% |
| POD0 (acute, non-SS) | Linear extension (last 2 points) | 80 | 140.0 [100.1–205.5] | 158.3 ± 75.6 | +0.8% |
| POD0 (acute, non-SS) | Flat (C12 = last sample) | 80 | 141.4 [101.0–207.5] | 159.8 ± 76.5 | +1.8% |
| POD0 (acute, non-SS) | Observed window only (no ext) | 80 | 123.7 [87.6–187.2] | 140.6 ± 67.5 | –10.9% |
| POD7 (reference, stable) | SS (C12 = C0, primary) | 80 | 105.4 [85.2–129.2] | 111.9 ± 43.6 | +0.0% |
| POD7 (reference, stable) | Linear extension (last 2 points) | 80 | 95.9 [78.3–121.1] | 103.6 ± 40.8 | –9.0% |
| POD7 (reference, stable) | Flat (C12 = last sample) | 80 | 105.6 [84.0–132.2] | 112.1 ± 44.7 | +0.2% |
| POD7 (reference, stable) | Observed window only (no ext) | 80 | 79.1 [63.7–99.6] | 85.8 ± 38.6 | –25.0% |

*Primary AUC0-12 used the steady-state assumption (C12 = C0). Alternative methods agreed within ±2% for POD0; observed-window only underestimates by 11% (POD0) and 25% (POD7) by omitting the 11→12 h interval.*

Abbreviations: POD, post-operative day; AUC0-12, area under the concentration-time curve over 0–12 h; IQR, interquartile range; SD, standard deviation; SS, steady state.

**Supplementary Table S2. Derivation of POD0 trough C0 target equivalent to the POD7 reference AUC0-12.**

| **Regression model** | **POD0 C0 for POD7 ref AUC median (125), ng/mL** | **POD0 C0 for POD7 ref AUC IQR (112-144), ng/mL** |
| --- | --- | --- |
| Linear regression (AUC = 59 + 12.0·C0) | 5.5 | 4.4–7.1 |
| Power model (AUC = 40.6·C0^0.63) | 5.9 | 5.0–7.4 |
| Median quantile regression (AUC = 36 + 13.0·C0) | 6.8 | 5.8–8.3 |
| Convergent estimate (range across models) | 5.5–6.8 | 4.4–8.3 |

*Reference AUC0-12 derived from POD7 patients with C0 in the conventional target range (7-9 ng/mL, n=26): median 125 [IQR 112–144] ng·h/mL. POD0 C0 estimates calculated by solving each regression equation for the reference AUC.*

Abbreviations: POD, post-operative day; C0, trough concentration; AUC0-12, area under the concentration-time curve over 0–12 h; IQR, interquartile range.

**Supplementary Table S3. AUC0-12 by trough C0 stratum (default 7/9 cutoff and sensitivity 5/10 cutoff).**

| **Period** | **Cutoff** | **C0 stratum** | **n** | **C0 median [IQR]** | **AUC0-12 median [IQR]** | **AUC0-12 mean ± SD** | **Kruskal–Wallis p** |
| --- | --- | --- | --- | --- | --- | --- | --- |
| POD7 (reference, stable) | Default (7/9) | <7 | 51 | 5.40 [4.85–6.25] | 93.4 [79.2–106.5] | 97.3 ± 33.5 | <0.001 |
| POD7 (reference, stable) | Default (7/9) | 7–9 | 26 | 7.85 [7.45–8.47] | 125.0 [111.8–143.8] | 127.5 ± 20.9 |  |
| POD7 (reference, stable) | Default (7/9) | >9 | 3 | 10.00 [9.65–11.30] | 185.7 [159.3–271.4] | 225.3 ± 117.3 |  |
| POD0 (acute, non-SS) | Default (7/9) | <7 | 35 | 4.70 [3.70–5.95] | 101.2 [79.3–152.2] | 121.9 ± 62.8 | <0.001 |
| POD0 (acute, non-SS) | Default (7/9) | 7–9 | 21 | 7.40 [7.20–7.80] | 131.2 [108.5–151.5] | 135.0 ± 40.5 |  |
| POD0 (acute, non-SS) | Default (7/9) | >9 | 24 | 15.10 [11.45–16.15] | 237.9 [188.0–277.0] | 232.4 ± 62.2 |  |
| POD7 (reference, stable) | Sensitivity (5/10) | <5 | 16 | 4.60 [4.15–4.80] | 76.7 [58.5–91.2] | 85.4 ± 45.0 | <0.001 |
| POD7 (reference, stable) | Sensitivity (5/10) | 5–10 | 63 | 6.90 [5.95–7.75] | 111.4 [94.6–131.0] | 114.8 ± 27.8 |  |
| POD7 (reference, stable) | Sensitivity (5/10) | >10 | 1 | 12.60 [12.60–12.60] | 357.2 [357.2–357.2] | – |  |
| POD0 (acute, non-SS) | Sensitivity (5/10) | <5 | 19 | 3.70 [3.10–4.15] | 85.3 [63.8–107.5] | 95.7 ± 43.1 | <0.001 |
| POD0 (acute, non-SS) | Sensitivity (5/10) | 5–10 | 39 | 7.10 [6.20–7.75] | 131.2 [101.2–171.3] | 143.6 ± 54.1 |  |
| POD0 (acute, non-SS) | Sensitivity (5/10) | >10 | 22 | 15.35 [11.65–16.25] | 252.0 [195.4–280.3] | 239.0 ± 59.7 |  |

*Default cutoff (7/9 ng/mL) followed the institutional target trough range. Sensitivity cutoff (5/10 ng/mL) was provided to assess robustness.*

Abbreviations: POD, post–operative day; AUC0–12, area under the concentration–time curve over 0–12 h; IQR, interquartile range; SD, standard deviation; SS, steady state.

**Supplementary Table S4. C0-adjusted comparison of POD0 versus POD7 AUC0-12 (ANCOVA).**

| **Matched trough C0 (ng/mL)** | **Predicted POD0 AUC0-12** | **Predicted POD7 AUC0-12** | **Difference (POD0 − POD7)** | **Relative difference** |
| --- | --- | --- | --- | --- |
| 6 | 131 | 104 | +27 | +26% |
| 7 | 143 | 121 | +22 | +18% |
| 8 | 155 | 138 | +17 | +12% |

*Predictions from an ANCOVA model (AUC0-12 ~ C0 × day; n = 80). The common-slope adjusted difference (POD0 higher than POD7 at the same C0) was +23.6 ng·h/mL (95% CI +9.3 to +38.0). At a matched trough, POD0 exposure remained significantly higher than POD7, although the difference was smaller than in the unadjusted comparison.*

Abbreviations: AUC0-12, area under the concentration–time curve over 0–12 h; C0, morning trough concentration; CI, confidence interval; POD, post-operative day.

**Supplementary Table S5. Matched-timepoint sensitivity analysis of POD0 AUC0-12 (three-point versus five-point sampling).**

| **POD0 sampling design** | **n** | **AUC0-12 median [IQR] (ng·h/mL)** |
| --- | --- | --- |
| Full (5 points: C0, C2, C3, C4, C10) | 80 | 145.4 [103.6–220.0] |
| Sparse (3 points: C0, C2, C10) | 80 | 174.3 [112.8–269.8] |

*The three-point AUC0-12 (matching the POD7 sampling density) slightly overestimated the five-point value (median within-patient difference +15.8%; IQR +10.2 to +20.1), reflecting the trapezoidal rule on the steep POD0 curve. Even at matched three-point density, POD0 AUC0-12 (174.3) remained far higher than POD7 AUC0-12 (105.4 ng·h/mL; Wilcoxon p < 0.001), confirming that the difference is not an artifact of the asymmetric sampling design.*

Abbreviations: AUC0-12, area under the concentration–time curve over 0–12 h; C0/C2/C3/C4/C10, concentrations at 0/2/3/4/10 h post-dose; IQR, interquartile range; POD, post-operative day.

**Supplementary Table S6. POD0 versus POD7 exposure stratified by pre-transplant tacrolimus exposure.**

| **Group** | **n** | **POD0 C0** | **POD0 AUC0-12** | **POD7 AUC0-12** | **Paired p** | **C0-adjusted difference (95% CI)** |
| --- | --- | --- | --- | --- | --- | --- |
| Longer pre-exposure (desensitized/ABO-incompatible) | 23 | 6.2 [5.0–7.2] | 129.6 [96.0–191.2] | 98.4 [85.2–124.9] | 0.06 | +17.0  (−15.6 to +49.5) |
| Shorter pre-exposure (standard recipients) | 57 | 7.7 [5.0–11.6] | 147.6 [102.3–210.5] | 107.9 [85.4–130.3] | <0.001 | +25.7  (+10.0 to +41.4) |

*Values are median [IQR] (ng/mL or ng·h/mL). Both groups showed the same direction (POD0 > POD7); the discordance was clearest in the shorter-exposure group. High POD0 troughs (C0 > 10 ng/mL) occurred predominantly in the shorter-exposure group (19 of 22), indicating that elevated POD0 exposure reflects early accumulation-phase pharmacokinetics rather than longer pre-transplant dosing. The C0-adjusted difference is from a common-slope ANCOVA (POD0 minus POD7 at the same C0).*

Abbreviations: ABO, ABO blood group; AUC0-12, area under the concentration–time curve over 0–12 h; C0, morning trough concentration; CI, confidence interval; IQR, interquartile range; POD, post-operative day.
